# Perceptions about the cultural practices of male partners during postpartum care in rural Tanzania: a qualitative study

**DOI:** 10.1080/16549716.2017.1361184

**Published:** 2017-09-08

**Authors:** Gladys Reuben Mahiti, Columba K. Mbekenga, Angwara Dennis Kiwara, Anna-Karin Hurtig, Isabel Goicolea

**Affiliations:** ^a^ Department of Development Studies, School of Public Health and Social Sciences, Muhimbili University of Health and Allied Sciences, Dar es Salaam, Tanzania; ^b^ School of Nursing and Midwifery, Agakhan University, Dar es Salaam, Tanzania; ^c^ Division of Epidemiology and Global Health, Faculty of Medicine, Umeå University, Umeå, Sweden

**Keywords:** Postpartum, practices, perceptions, Tanzania, male partners

## Abstract

**Background**: Men play an important role in maternal health. The postpartum period is a critical stage, yet there is a scarcity of research that explores men’s involvement during this stage.

**Objective**: The aim of the study was to explore male partners’ perceptions of the cultural practices during postpartum care in rural Tanzania.

**Methods**: Fourteen focus group discussions were conducted with 93 men, with an age range of 19–65 years, in August 2013. The study was conducted in the Kongwa District, located in the Dodoma region in central Tanzania. Qualitative data were digitally recorded, transcribed verbatim and analyzed using content analysis.

**Results**: Four categories emerged, namely: ‘Men as providers and, occasionally, care takers’, ‘Men as decision makers’, ‘Diverse perceptions of sexual abstinence’ and ‘Barriers for men in using/accompanying partners to use reproductive and child healthcare services’. The cross-category theme ‘Men during postpartum: remaining powerful but excluded’ refers to how men are in a powerful position above women in different aspects of life. Elderly women played an important role in caring for postpartum mothers and their newborns, but men were the ones making the final decision about where to seek care. Traditional practices isolated men from their partners for a certain period, and enforced sexual abstinence for the women during the postpartum period. However, cultural norms permitted men to engage in extramarital relations. Reproductive and child healthcare services were perceived by men as not welcoming the male partners, and local gender norms discouraged men from accompanying their partners to seek services.

**Conclusions**: In this study, we found that men perceived their role during the postpartum period as financial providers, decision makers and, occasionally, care givers. Men also held diverse perceptions with regard to sexual abstinence and felt excluded from participating in maternal healthcare services.

## Background

Men play an important role in women’s and children’s health [1–3]. Studies have primarily focused on the negative impact of men’s behavior on women’s health, e.g. violence, unwanted pregnancy and human immunodeficiency virus (HIV) infection [4–7]. However, men can also play a positive role, and men’s involvement in pregnancy and childbirth has been shown to benefit the health of mothers and newborns [1,8–13]. Men’s involvement in reproductive health can also enhance gender equality and empower individual women to make their own decisions [14,15]. Men as individuals can also benefit from being involved in parenting and reproductive health programs [16]. However, it is important to point out that men’s increased involvement in reproductive health programs could also lead to men having increased control over women’s reproductive choices [17].

Despite the potential that men’s involvement in maternal healthcare has in terms of improving the health of the men, women and children, men’s actual involvement remains low in most African countries [1,17–20]. To understand why, it is necessary to consider gender relations [20]. The way in which gender relations are constructed in each particular setting and time influences every aspect of people’s life, including reproductive decisions, the provision of care and the utilization of healthcare services [20–23]. According to Connell, poor participation of men in reproductive and maternal health is the result of gender structures that strongly influence individual men’s decisions. Such gender structures encompass the sexual division of labor and power, and emotional and symbolic relations. The literature shows that gendered norms and expectations, in general, hinder men’s involvement in reproductive healthcare and their access to programs and services [24–26]. However, there are also studies pointing out that social norms exist that may promote men’s involvement in reproductive health [26–28]. Some studies in sub-Saharan Africa also revealed that men are often bound to normative social and cultural contexts fueled by gender expectations [13,29–31].

In Tanzania, reproductive and child health policies and guidelines acknowledge the importance of promoting men’s involvement during all the stages of the continuum of maternal and newborn care [18,32–35]. While there has been an increase in men’s involvement in antenatal care services, especially in prevention of mother-to-child HIV transmission programs, their involvement in postpartum care remains low. The postpartum period tends to be the most neglected stage in maternal care, despite the opportunity that it provides to detect complications, to promote reproductive and child health, to promote nutrition and provision of vitamin A supplementation, and to complete tetanus toxoid immunization if required [34]. The Tanzanian postpartum guidelines advocate the importance of involving men during this period, but offer little information on how to implement this [35].

There is a scarcity of research exploring men’s practices and perceptions during the postpartum period both in Tanzania and elsewhere. Existing research has been conducted in urban areas and focused mainly on breastfeeding and prevention of mother-to-child HIV transmission [35–38]. The situation in rural areas, where the implementation of guidelines may be more erratic and the influence of social norms hindering men’s involvement may be stronger, has not been explored. The aim of this study was to explore male partners’ perceptions about the cultural practices during postpartum care in rural Tanzania.

## Methods

### Study area

The study was conducted in the Kongwa district of the Dodoma region, located in central Tanzania. The Kongwa district has a population of 248,656, 90% of whom live in rural areas. The main economic activity of the population in this area is agriculture, including crop cultivation and livestock keeping. People in this area also engage in other activities, such as trade and small-scale mining. The transport infrastructure is limited and existing roads are in poor condition, making it difficult to access healthcare services. The district was purposively selected because of its rural characteristics and the ongoing research focused on health systems.

According to 2010 statistics, 97.4% of women in mainland Tanzania receive at least one antenatal care visit, while 58.3.5% receive two or three visits, and 39.1% receive four or more visits. Delivery takes place at healthcare facilities in the Dodoma region in 45.9% of cases, and only 33.8% of mothers receive postpartum check-ups, defined as check-ups of the mother and the child [39].

Districts are divided into wards, villages, hamlets and households. Three villages were included in this study: Kongwa, which is a peri-urban village, and Ibwaga and Ugogoni, both located in remote places.

There are few indicators available that provide insight into gender relations and women’s status in the Dodoma region. The Tanzania Demographic and Health Survey (TDHS) 2010 indicated that about 48.5% of women in the Dodoma region were unable to make their own decisions in terms of their health, household purchases and visits to their relatives, and 68.9% of women had experienced intimate partner violence in their lifetime [39]. The TDHS does not currently include any information on men’s involvement in reproductive health and/or maternal healthcare services.

### Study design and data collection

This study used a qualitative design to explore men’s perceptions about the cultural practices during postpartum period. Data collection and analysis were done in an interactive way, and any new, unexpected findings that emerged were incorporated in the process [40]. Qualitative research is grounded on the assumption that reality is subjective, multiple, socially constructed and contextually bound [40].

Focus group discussions (FGDs) were chosen as the method of data collection because they facilitate enhanced interaction between participants, and provide an opportunity to observe social and cultural norms [41].

Data collection was carried out in August 2013. Purposive sampling was used to select the villages and participants, meaning that participants were selected based on their experience of having postpartum partners or a baby born within 5 years of the study. Participants were approached through the guidance of village and ward leaders. The researcher explained the objective of the study for them before asking their participation, and asked their permission to audio-record the FGD. The FGDs were conducted in the local language of Kiswahili. The first and second authors, who are fluent in the language, moderated the discussions. The first author moderated all the FGDs, while the second author acted as an observer for some of the FGDs. In all FGDs a male research assistant was also present who took notes in relation to the participants’ responses and interaction. Debriefing sessions between the moderator, observer (when present) and note-taker took place after every FGD. The FGDs took place in the village or ward offices, depending on the preference of participants.

Data were collected through 14 FGDs with a total of 93 men. The participants’ age ranged from 19 to 65 years. The majority of the participants (71%) had completed primary education (standard seven) and most of them (89%) were engaged in agricultural activities. Data collection stopped when a saturation point was reached, i.e. no new information related to our research question was emerging [40].

The FGDs gathered information on the following issues: roles that men play in the postpartum period, their perspectives regarding maternal care during the postpartum period, cultural traditions related to the postpartum period and sexual relations. They also explored their participation in formal postpartum care services and their perceptions on women’s use of formal postpartum care services.

### Data analysis

The digitally recorded FGDs were transcribed verbatim and entered into Open Code 3.4 to manage the data and facilitate the analysis [42]. Data were analyzed using qualitative content analysis [40]. First, the FGDs were read several times to obtain a sense of a whole, and to identify meaning units, i.e. short sections of the transcripts that were meaningful and related to our research question. Secondly, the identified meaning units were condensed into short summarized versions, what Graneheim and Lundman [40] call condensed meaning units. Thirdly, from the condensed meaning units, codes were further elaborated. Fourthly, codes were grouped together, and through going back and forth between the text and the developed codes and preliminary groups, categories were developed. Categories refer to a higher level of abstraction, but still convey the manifest content of the transcripts. Finally, through constant comparison between categories and the rest of the material, a theme was constructed that cut across the categories and reflected the latent content of the text [40]. (See Table A1 for an example of the process followed in the analysis.) Coding was conducted with the Kiswahili transcripts to remain close to the text. Codes were then translated into English to develop categories and themes. Among the researchers, three are native Swahili speakers.

## Results

In this study, four categories emerged, namely: ‘men as providers and, occasionally, care takers’, ‘men as decision makers’, ‘diverse perceptions of sexual abstinence’ and ‘barriers for men in using/accompanying partners to use reproductive and child healthcare services’. Later, a theme emerged that cut across the categories: ‘men during postpartum: remaining powerful but excluded’. The theme refers to how men were in a position of power above women in different aspects of life, including regarding decision making on healthcare issues. However, the postpartum period was a particular time when men were, to a certain extent, excluded from care, both at home and in the healthcare facilities. Elderly women and older children played a more important role in caring for postpartum mothers. Traditional practices isolated men from their partners for a certain period and enforced sexual abstinence, and healthcare services were perceived by men as not welcoming them (see Table A2).

### Men as providers and, occasionally, care takers

Participants in this study expressed that their main responsibility postpartum was to provide for the family’s needs, including the mother, supporters and the baby.

‘Well! It’s true that the husband is responsible for other activities such as income-generating activities. […] The husband is expected to provide for the family since there is expenditure every day. So, a husband must continue to produce to cater for household expenditure.’ (FGD number 6)

Participants were of the view that their partners had to rest from working during the postpartum period, and that they should not be engaged in household chores for a certain period postpartum. Men reported that support for the postpartum mother was mainly provided by other women. Such support involved taking on the household chores, such as cooking, but also more intimate acts, such as bathing the postpartum mother. Male partners might take on some of the household chores, such as fetching firewood, fetching water and cultivating land. They might engage in caring for the postpartum mother if other supporters were missing. However, in general, men expressed that they were excluded when it came to taking care of the postpartum mother. Some men expressed that they had been excluded to the extent that they did not know what was going on concerning the care of the postpartum mother.

‘The way I am used to see[ing] is that, normally there are people who assist in doing some trivial chores once a woman gives birth. Maybe a woman will have a rest for one week … eeeh … that’s how we are used to see[ing]. She will slowly start to take over her normal chores once she feels a bit recovered …. eeeeh at the same time, as a husband, you will have to assist in other chores such as fetching water, firewood eeeh … that’s how we normally do it here.’ (FGD number 5)

### Men as decision makers

Men in this study reported that they made decisions in the household as they recognized themselves as being in charge and the head of the household. Participants reported that they encouraged women to use healthcare services. It was observed that a man’s position as head of the household gives him the ability to control his partner. Men also reported that the elders who were caring for their partner during the postpartum period would consult them, for example, when their partners became sick. As the elders are the ones who have been close to the postpartum mothers, it was easier for them to notice any problem that may arise. On these occasions, men were expected to make decisions in terms of whether or not to seek care from formal healthcare facilities, and when to do it. However, participants also reported that this was a period when other people may hold decision-making power. The elderly women also had a role in the decision as they had been taking care of the postpartum women, which helped them to identify the problems.

‘It is not possible for a mother to decide not to go to the hospital. As for me as head of the household I instructed her that I need to bring her to the hospital. I do not think she can refuse to go to the hospital.’ (FGD number 12, Men)

### Diverse perceptions of sexual abstinence

Participants in this study expressed that they slept in a separate room during the postpartum period, as they were supposed to abstain from having sex with their partners. Sexual abstinence postpartum was considered to be rooted in tradition and strongly supported and controlled by the elderly women who usually took care of the postpartum mother. Participants described how they should abstain for three months if they had a baby boy and for four months if they had a baby girl. Having sex with their partners before that put the baby at risk of ‘kumtima’. Kumtima is a Gogo term that describes the health problems, including diarrhea and weight loss, that the baby will face if his or her parents engage in sex too early.

‘That tradition is about customs of the Gogo tribe. If a woman delivers a baby boy, she will not be able to have sex with husband for three months eeeh … If a woman has delivered a baby girl, you will be allowed to have sex with your husband after four months eeeh. More patience is needed for that one since it is a tradition among Gogo tribe. We are taught that a man should restrain himself from having sex with any kind of woman for three months after your wife has delivered.’ (FGD number 5)

Despite the powerful influence of this tradition, men also reported that there were ways to get around it. Participants considered that men’s sexual drive could not be controlled, and consequently men had to find ways to have sex without risking the health of their babies. Having sex with other women was considered a commonly used alternative.

‘Regarding the issue of extramarital relations, I was told by my grandmother that, if my wife has delivered a baby its normal to have extramarital sexual intercourse. Thus when I come back home, there is no possibility of harm to the newborn. With extramarital sex, its normal to do that and once you come back to your wife, it is just business [resuming sex] as usual … eeeh.’ (FGD number 7)

### Barriers for men in using/accompanying partners to use reproductive and child healthcare services

Participants pointed out several factors that inhibited them from accompanying their partners to the reproductive and child health clinic during the postpartum period. First, they felt shy as they thought that it was a ‘women’s business’ to use reproductive and child healthcare services. In addition, it was not common in the community for a man and his partner to attend the reproductive and child health clinics together.

‘We the males are proscribed from using the places used for women[’s] gatherings.’ (FGD number 12)

Secondly, participants considered that if a man accompanied his partner to the healthcare service, he might risk being perceived as being ‘controlled’ by his partner and may be criticized or teased.

‘A man has a feeling that he is controlled. It is not common in the Gogo traditions to go to health facilities with women, so men are fearful to accompany their partners’. (FGD number 11)

Thirdly, the formal healthcare system does not advocate men’s use of reproductive healthcare services during the postpartum period, or at any other point. Participants expressed that the health system does not make men aware of the importance of using reproductive and child healthcare services. Furthermore, the men elaborated that the availability of only female health workers delivering reproductive education hindered them from using reproductive and child health services because they felt uncomfortable.

‘Nowadays you find a woman [female nurse/midwife] coming to educate us on reproductive issues thus attracting poor enrollment of men in these programs.’ (FGD number 12)

When asked about the importance of using postpartum care services, participants in this study considered that their role was more relevant during pregnancy, by escorting mothers for HIV testing. Participants expressed that the benefits of reproductive and child healthcare services were mainly for the baby and they were intended for immunization and weight monitoring.

They did not consider that postpartum care was also of benefit for the mother, unless complications emerged.

‘Eeee, she must go back since in some occasions, the baby will need some drops for vaccination. That’s why they normally go back.’ (FGD number 6)

‘To me, here in our places, if the mother got complications, we normally rush her to the health facility. We do that for the woman to get treatment, eehmm.’ (FGD number 5)

## Discussion

The study found that during the postpartum period men perceived their main role as providing for the family financially. While occasionally they could take on caregiver roles, if other caregivers were not available, they mainly engaged in ‘masculine’ tasks that involved physical strength. During the postpartum period, their role as the main decision makers might be challenged by the key role that women, especially elderly women, played during that period. Traditions that promote sexual abstinence postpartum limited men’s sexual access to their partners. Extramarital sexual relations were considered as a way to deal with the prohibition of sexual relations with the postpartum mother for a certain number of months after delivery. Finally, men faced barriers to using or accompanying their partners to reproductive and child healthcare services, both due to cultural norms that do not encourage men to access services and/or be involved in maternal and child care, and due to the fact that women are still the main target of healthcare services.

Participants’ perception on their role during the postpartum period were closely connected with gender aspects. Connell describes gender as something constructed through social relations with others [21]. She describes three interconnected dimensions of gender relations: power relations – how power is exercised, for instance through violence, dominance or control; production relations – the way gender influences work and the division of labor; and cathexis – which includes worldviews and emotions [21,22].

According to Connell, men can practice power in different ways: via controlling, authority, coercion and/or violence [21]. In this study, men’s power and dominance were observable in the way that men had the final say in deciding when women should seek care from formal health facilities. Even if other women were the ones noticing postpartum health problems, it was still up to the husband or partner to decide whether a woman could seek healthcare or not, which might lead to delays in seeking care.

Previous studies in many countries, including Tanzania, show similar results, namely women depending on their male partners to make decisions on whether and where to seek medical care, which limits women’s right to sexual and reproductive health [17,18,38,41,43,44]. However, this prevalent position is not universal [34].

The way in which sexual relations were constructed also reflected men’s power over women in this study. According to participants, it was acceptable for men to have extramarital relations as a way of avoiding ‘kumtima’, while the same did not apply to women. Other studies also show double sexual standards for women and men, and how sexual relations are strongly connected with the display of dominance and the maintenance of women’s subordination [45–48]. Such practices may also increase the risk of acquiring sexually transmitted infections and HIV. However, it is important to notice that sexual abstinence during this period can also have positive effects for the woman, i.e. it allows for proper healing of vaginal injuries from the delivery and is effective for child spacing [49–53].

This study shows that the postpartum period is a time when men lose certain decision-making powers and have more limited access to their wives. By constructing the postpartum period as the ‘domain’ of women, men became excluded. The fact that certain issues and periods are considered ‘feminine’, instead of stereotyping and disempowering women, may contribute towards empowering them, for example by leaving women to control all activities involving postpartum mothers and having an influence in the decision to seek care. Findings from another study in Tanzania also supported the relevant role of women, especially elderly women, during delivery and postpartum [18]. However, as other authors point out, when men lose power in certain areas they may try to compensate for this temporarily disempowered position by exercising power in more explicit ways, such as through violence against women [54,55].

According to Connell, production relations refer to the way in which gender influences work and the division of labor [21]. The gendered division of work postpartum became very explicit in this study. Men were in charge of providing for the family and they were expected to fulfill this role during the postpartum period, as well as at other times. Our findings are similar to those of other studies, which show that men are the main breadwinners and women depend on them for economic support [38,48,56,57]. This study also points out that men were not the most involved in ‘caring’ and household work while the postpartum mother was convalescing. Other members of the extended family, mainly women, would be called in to take on these chores. These findings differ from other studies, which found that the postpartum mothers themselves had to take on these chores [39,58].

Cathexis refers to gendered social and cultural norms and emotional relations [21,23]. The gendered cultural norms in Kongwa district hindered men’s engagement with caring for the mother and child. This may have harmful effects for the baby and the mother, and also for the father, who may feel excluded from a key life event. Similar research elsewhere found that excluding men from caring for their partners led to their feeling confused, helpless, anxious and useless, which can create unhealthy couple relationships [10]. Similar findings were also reported elsewhere in Tanzania and other parts of the world [37]. Cultural norms also affected access to healthcare services. In Kongwa, as elsewhere, healthcare services, especially maternal healthcare services, focus on women, and men are hardly involved owing to cultural issues. This findings are also in line with similar findings from sub-Saharan Africa, that men are often bound to normative social and cultural contexts fueled by gendered expectations [13,29–31]. Health professionals do not expect them to come, do not encourage them and may have negative perceptions of men’s involvement [13,25]. Numerous studies have also indicated the barriers that men face in accessing health services for themselves and becoming engaged with services during pregnancy and childbirth [1,26,34,38]. Policies and programs should take into consideration these societal barriers if they want to enhance men’s engagement during this period.

### Trustworthiness of the study

Criteria used to evaluate the trustworthiness of qualitative research include credibility, dependability and transferability. Credibility refers to the ability to capture the multiple perspectives under study. We used a number of strategies to enhance the credibility of the study. First, the first author spent periods in the field to become familiar with the setting. Secondly, triangulation was done at the level of the researchers involved in the study, who brought different perspectives and had different degrees of familiarity with the setting. Dependability is defined as the possibility of changing the data throughout data collection and analysis and the ability of the researcher to consider this. Transferability is how applicable the findings are to other contexts [59].

Two married women researchers conducted the FGDs with the men. This may have influenced the participants’ answers, i.e. they may have been more polite and less prone to disclose certain practices such as extramarital sex. However, the participants seemed relaxed during the discussions, and participation was very active. The attitudes of the two researchers and the presence of a male research assistant may have facilitated the generation of an enabling environment for the discussions. Men from different age groups were included in the FGDs to increase the variety of perspectives. However, we acknowledge that having both young and older men could have led to the younger men not felling free to express their opinions or to disagree with the older men.

The transcripts were initially analyzed in the Kiswahili language, which allowed us to stay close the participants’ original language in identifying the meaning units and codes. Translation of the codes to English, and discussion of emerging subcategories, categories and theme among researchers in the team enriched the interpretation of the data through the balance of perspectives representing different backgrounds and qualitative expertise. These measures enhanced the credibility of the representation of participants’ views that is presented in our findings.

The detailed description of the study context, selection criteria, data collection and analytical process was complemented by quotations to allow readers to judge the dependability of the analysis and transferability of the findings.

### Conclusions

In this study, we found that men perceived their role during the postpartum period as financial providers, decision makers and, occasionally, care givers. Men also held diverse perceptions with regard to sexual abstinence and felt excluded from participating in maternal healthcare services.

Cultural practices in relation to sexual relations are gendered, and so are cultural practices related to the postpartum period. Healthcare institutions are also gendered and display different practices when treating men and women. In this study, the role of men during the postpartum period is highlighted, both the fact that certain cultural practices rooted in gender inequality threaten women’s health, and the fact that men are excluded and held unaccountable during this period. An important and long-term implication of this study is the need to challenge gender inequality to improve women’s health postpartum. Another implication, which should be more attainable in the short term, is the need to make healthcare systems more inclusive towards men during pregnancy, delivery and the postpartum period. Such interventions are now promoted by international organizations, but it is key to consider cultural contexts when designing them. One-size-fits-all interventions to encourage men’s participation during the postpartum period will not work if they fail to consider cultural specificities. This study uncovers some of the cultural practices in rural Tanzania that should be taken into consideration when designing such interventions in this setting.

## Paper context

The role of men in maternal health is receiving increased attention, but there are scarce studies focusing on the postpartum period in rural low-income settings. This study found that men in rural Tanzania perceived their role postpartum as financial providers, decision makers and occasionally care givers, while they felt excluded from participating in maternal healthcare services. Policies for improving the coverage and quality of care during the postpartum period should aim for promoting men’s involvement.

## Appendix

**Table A1.** An example of the coding process leading to the category ‘Men as providers and, occasionally, care takers’.

Meaning unit	Condensed meaning unit	Selected codes	Subcategories
When the postpartum mother comes back home, for us always there is her mother and my mother if she is alive. They might be there for a week caring but they cannot leave until the mother has enough energy as I go away to work and look for food, but when they go back, I remain alone taking care of her	When postpartum mother comes back home, her mother and my mother care for her. I go away to work and look for food. When they leave I remain alone taking care of her	Postpartum care at home Female relatives in charge of postpartum care Elders take care of postpartum mother Man goes away to work Man goes away to look for food Man caregiver when elders go away	Postpartum care support as women’s work Men participate in income-generating activities Men provide care postpartum

**Table A2.** Categories and subcategories referring to practices and perceptions of male partners in the postpartum period.

S/N	Categories	Subcategories
1.	Men as providers and, occasionally, care takers	Men provide care during postpartum Men participate in income-generating activities Postpartum care support as women’s work
2.	Men as decision makers	Men decide when to seek care Elderly women provide information to the male partner concerning a mother’s sickness Men as head of the household
3	Diverse perceptions of sexual abstinence	Men sleeping in another room within the household Extramarital relations as coping strategy during postpartum period Couples have sexual abstinence for three or four months depending on child’s gender
4.	Barriers for men in using/accompanying partners to use reproductive and child healthcare services	Society believes that women are controlling husband if he goes to healthcare services Lack of advocacy on male involvement from the healthcare system Female health workers giving reproductive health education make men feel shy Postpartum care services perceived to be of use for the baby and if complications to mothers arise
